# Engagement challenges in digital mental health programs: hybrid approaches and user retention of an online self-knowledge journey in Brazil

**DOI:** 10.3389/fdgth.2024.1383999

**Published:** 2024-09-25

**Authors:** Felipe Moretti, Tiago Bortolini, Larissa Hartle, Jorge Moll, Paulo Mattos, Daniel R. Furtado, Leonardo Fontenelle, Ronald Fischer

**Affiliations:** ^1^ Cognitive and Neuroinformatics Unit, D’Or Institute for Research and Education, São Paulo, Brazil; ^2^ Cognitive and Neuroinformatics Unit, D’Or Institute for Research and Education, Rio de Janeiro, Brazil; ^3^ Neuroscience Unit, D'Or Institute for Research and Education, Rio de Janeiro, Brazil; ^4^ Open D'Or Unit, D'Or Institute for Research and Education, Rio de Janeiro, Brazil

**Keywords:** mental health, mental telehealth, self-knowledge, self-care, telepsychiatry

## Abstract

Digital mental health interventions (DMHIs) have surged in popularity over the last few years. However, adherence to self-guided interventions remains a major hurdle to overcome. The current study utilized a phased implementation design, incorporating diverse samples and contexts to delve into the engagement challenges faced by a recently launched online mental health platform in Brazil with self-evaluation forms. Employing an iterative mixed-methods approach, including focus groups, online surveys, and think-aloud protocols, the research aims to evaluate user satisfaction, identify barriers to adherence, and explore potential hybrid solutions. Engagement in the platform was evaluated by descriptive statistics of the number of instruments completed, and qualitative interviews that were interpreted thematically. In the fully self-guided mode, 2,145 individuals registered, but a substantial majority (88.9%) engaged with the platform for only 1 day, and merely 3.3% completed all activities. In another sample of 50 participants were given a choice between online-only or a hybrid experience with face-to-face meetings. 40% of individuals from the hybrid group completed all activities, compared to 8% in the online-only format. Time constraints emerged as a significant barrier to engagement, with suggested improvements including app development, periodic reminders, and meetings with healthcare professionals. While the study identified weaknesses in the number and length of instruments, personalized results stood out as a major strength. Overall, the findings indicate high satisfaction with the mental health platform but underscore the need for improvements, emphasizing the promise of personalized mental health information and acknowledging persistent barriers in a digital-only setting.

## 1 Introduction

Digital mental health interventions (DMHIs) have surged in popularity, especially in the wake of the COVID-19 pandemic, partly because they expand much-needed access to healthcare services (1, 2) and their cost-effectiveness (3). However, a major research gap to overcome in using digital health programs is user adherence (4). This issue is particularly acute in low- and middle-income countries (LMICs) (5). A scoping review of internet-based interventions for the prevention and treatment of mental health disorders in Latin America identified that most studies were conducted in Brazil, with one of the results highlighting difficulties in engaging and retaining participants for a long enough period (5). While there seems to be a general trend toward improved adherence when a human presence is involved in DMHIs, meta-analyses have not found statistically significant effects (6).

Recent studies demonstrated the relative effectiveness of unguided (self-help) digital interventions compared to guided interventions, but adherence remains a major hurdle (7). In unguided interventions, individuals typically drop out after one or two digital sessions (2), completing less than half of the online activities (6). Research examining data from 100,000 users of mental health apps revealed an average retention period of 5.5 days in the solutions, with most users discontinuing their usage after only 2 days (8). Nevertheless, when clinicians recommend some apps to patients as part of the study the average retention time increases significantly to around 40 days (8). Other studies also found lower adherence without human support (1, 9).

We present preliminary results of a digital health service in a context with significant needs. “The Online Self-Knowledge Journey” offers a self-guided digital education experience to enhance self-awareness and mental health in Brazil. This platform provides personalized feedback on key psychological factors as a core element of mental health education (10, 11) and as a form to engage users (12). In an earlier meta-meta-analysis examining self-guided mental health interventions, specific psychoeducational techniques like mindfulness, positive psychology, cognitive-behavioral approaches, acceptance and commitment methods, and activity-based interventions with various components such as relaxation, music, and physical exercise displayed promising effectiveness (11). Consequently, we integrated these elements, along with personalized feedback, into the Online Self-Knowledge Journey developed. Our objective is to evaluate the platform's user engagement and satisfaction in an understudied context. Using a mixed-methods design, we identify actionable strategies to improve user adherence and satisfaction in Brazil and other LMICs.

## 2 Methods

### 2.1 Development of the online self-knowledge journey

The self-knowledge journey grew out of an earlier mental health information service that attracted significant interest during the COVID-19 pandemic. It is a web-based platform allowing users to complete self-report validated psychological measures and receive personalized feedback with videos demonstrating evidence-based practices for enhancing mental health and wellbeing (10, 11). The journey contained five thematic areas (personality, values, well-being, relationships, and consciousness). At each area, validated psychological scales were presented, which, when answered, provided personalized feedback to participants in the form of graphics and texts describing individual results in relation to normative samples of the Brazilian population and following standardized psychometric measures (see an example in Supplementary Figure S1).

The feedback was produced with the aim of increasing users' self-knowledge, self-awareness, and engagement. The validated measures spanned various domains (see the full list of instruments and constructs in the OSF link). They were selected because of their prominence in the literature, safety, and information value for personal development. For example, the Generalized Anxiety Disorder 7-item (GAD-7), the Patient Health Questionnaire-9 (PHQ9), and the Big Five Inventory are the most established and widely used instruments to assess depression, anxiety, and personality traits (13, 14). The instruments were adapted for the Brazilian population and personalized feedbacks were calculated in real-time via the FormR framework (15). Access was free, but users had to register with a login ID and password. The development of the current version involved several iterations refined through user feedback (16). We evaluate overall satisfaction and identify barriers to user adherence using five samples and complementary methods.

### 2.2 Samples

The study employed a phased implementation strategy involving various samples and contexts to continuously improve the digital solution and gain a deeper understanding of the challenges associated with engaging users. Initially, the implementation occurred within small participant groups to identify potential technical glitches in more manageable settings. Additionally, this phase aimed to glean insights into user engagement challenges. Subsequently, we extended access to the solution to the research institution's employees to assess potential technical issues on a broader scale. Following improvements in technological robustness, we made the solution available to internet users. Upon encountering engagement hurdles during public release, we ran an exploratory pilot study with a new sample to compare the user engagement in online-only or a hybrid experience with face-to-face meetings. This phased implementation strategy involving different samples followed the order shown below:

**Sample 1:** Nine adults enrolled at a technical college in a medium-sized city in São Paulo, Brazil, participated in in-person focus group discussions over four weeks. Participants were encouraged to vocalize their thoughts as they navigated through the journey, highlighting challenges encountered and focusing on both positive and negative aspects of the experience. The sessions were recorded, transcribed, and analyzed to assess satisfaction and engagement. After the fourth session, participants responded to an online satisfaction survey (see Section 2.3.1).
**Sample 2:** Students and professors from a public university in São Paulo participated in an in-person relaxation and self-knowledge workshop (*N* = 18). Participants completed the first instrument available on the platform at the end of the workshop and were encouraged to continue it at home. After 2 weeks, the same online satisfaction survey (see Section 2.3.1) was sent to six participants who agreed to be re-contacted in the consent form.
**Sample 3:** Employees of a nonprofit research institute in Rio de Janeiro were invited via email to register on the platform. Of a total of 340 individuals contacted, 30 individuals registered. Three reminders were sent over a 3-week period. An online satisfaction survey (see Section 2.3.1) was sent to 25 individuals who agreed to be contacted. Think-aloud interviews were conducted with a sub-sample (*N* = 7).
**Sample 4:** The platform was publicly available in August 2023 and promoted via digital health and wellness websites. We analyzed data from registered users from August 2nd to November 2nd, 2023 (*N* = 2,145). Think-aloud interviews were conducted with a sub-sample of subjects who agreed to be re-contacted and responded to our invitation (*N* = 2) (see Section 2.3.2).
**Sample 5:** A group of 50 students from a private college in São Paulo participated and could self-select into either the online-only experience (*N* = 25) or the online experience with an additional face-to-face group meeting (*N* = 25). After 2 weeks, an online survey about facilitators and barriers to adherence (see Section 2.3.3) was sent to those who consented to have their data used for research purposes.

For demographics of all samples, see Table 1. For a timeline of all sample's interactions with the self-knowledge journey, see Supplementary Figure S2.

**Table 1:** Demographic information for each sample.

	Sample 1, *N* = 9^a^	Sample 2, *N* = 18^a^	Sample 3, *N* = 30^a^	Sample 4, *N* = 2,145^a^	Sample 5, *N* = 50^a^
Age	25 (20, 36)	23 (22, 35)	37 (35, 40)	30 (23, 40)	23 (20, 29)
Unknown	0	15	9	1,794	26
Sex					
Male	5 (56%)	3 (100%)	2 (9.5%)	105 (30%)	6 (25%)
Female	4 (44%)	0 (0%)	19 (90%)	245 (70%)	18 (75%)
Other	0 (0%)	0 (0%)	0 (0%)	2 (0.6%)	0 (0%)
Unknown	0	15	9	1,793	26
Education					
Elementary School	0 (0%)	0 (0%)	0 (0%)	12 (3.4%)	0 (0%)
Incomplete High School	1 (11%)	0 (0%)	0 (0%)	21 (6.0%)	0 (0%)
Complete High School	4 (44%)	1 (33%)	2 (9.5%)	66 (19%)	4 (17%)
Incomplete Graduation	1 (11%)	1 (33%)	0 (0%)	71 (20%)	18 (75%)
Complete Graduation	3 (33%)	0 (0%)	3 (14%)	58 (16%)	1 (4.2%)
Incomplete Post-Graduation	0 (0%)	0 (0%)	0 (0%)	41 (12%)	1 (4.2%)
Complete Post-Graduation	0 (0%)	1 (33%)	16 (76%)	83 (24%)	0 (0%)
Unknown	0	15	9	1,793	26
Race					
White	6 (67%)	3 (100%)	11 (52%)	191 (56%)	12 (50%)
Black	1 (11%)	0 (0%)	2 (9.5%)	30 (8.7%)	5 (21%)
Brown	2 (22%)	0 (0%)	7 (33%)	118 (34%)	7 (29%)
Indigenous	0 (0%)	0 (0%)	0 (0%)	2 (0.6%)	0 (0%)
Other	0 (0%)	0 (0%)	1 (4.8%)	0 (0%)	0 (0%)
Don't know/prefer not to inform	0 (0%)	0 (0%)	0 (0%)	3 (0.9%)	0 (0%)
Unknown	0	15	9	1,801	26
Income					
Well above the country's average	0 (0%)	0 (0%)	2 (9.5%)	54 (15%)	0 (0%)
Slightly above the country's average	0 (0%)	3 (100%)	5 (24%)	98 (28%)	3 (13%)
In the country's average	6 (67%)	0 (0%)	11 (52%)	126 (36%)	14 (58%)
Slightly below the country's average	2 (22%)	0 (0%)	2 (9.5%)	57 (16%)	6 (25%)
Well below the country's average	1 (11%)	0 (0%)	1 (4.8%)	17 (4.8%)	1 (4.2%)
Unknown	0	15	9	1,793	26

^a^ Median (IQR); *n* (%).

### 2.3 Measures and procedures

#### 2.3.1 Online satisfaction survey

Participants from samples 1–3 who consented to be re-contacted (*N* = 40) received an electronic survey to evaluate their overall satisfaction with the self-knowledge journey. Twenty participants responded to the survey. The following items were answered on 1 to 5 Likert-type (responses from “strongly disagree” to “strongly agree”, unless noted otherwise): (1) “The journey seems useful for improving wellbeing”; (2) “The journey seems beneficial for personal development”; (3) “I found the size or time to complete the questionnaires” (ranging from unsuitable to suitable); (4) “I found the number of questionnaires (ranging from unsuitable to suitable)”; (5) “I appreciated the visual and graphic design of the journey”; (6) “Overall, I rate the personalized feedback provided” (ranging from “poor” to “good”); (7) “The relevance of psychoeducational materials, such as videos and texts” (ranging from irrelevant to highly relevant). A multiple-choice option asking for reasons for discontinuation was also included.

#### 2.3.2 Focus group and think-aloud interviews

In the focus group meetings (sample 1), participants were encouraged to share their thoughts and feelings as they progressed through the self-knowledge journey. The group shared points of interest, difficulties, and demotivating elements. The think-aloud interviews (15) were conducted with nine participants (*N* = 9) from samples 3 (*N* = 7) and 4 (*N* = 2) to delve deeper into issues related to adherence. Participants were asked to recall their experiences, to share the motivators that got them to start the journey, and what discouraged them from continuing. The data on engagement from the focus groups and the think-aloud interviews were analyzed together.

#### 2.3.3 Online survey on facilitators and barriers to adherence

This survey was sent to consenting participants of Sample 5 (*N* = 44). Four multiple-choice questions with more than one answer option elicited reasons for stopping the journey, suggestions for improvement, and strong and weak points of the platform. The following questions were rated on five-point Likert-type scales: (1) How interested are you in topics like mental health and self-knowledge?’ (“no interest” to “very interested”); (2) “The experience I had was” (“bad” to “very good”); (3) “I learned relevant things” (“strongly disagree” to “strongly agree”); (4) “I found the personalized results” (“bad” to “very good”); (5) I found the number of questionnaires (“unsuitable” to “suitable”); (6) My chance of recommending the journey to someone I know is (“very low” to “very high”).

### 2.4 Data analysis

Descriptive statistics are reported for the online satisfaction surveys (Samples 1–3) and facilitators and barriers to adherence survey (Sample 5). We analyzed engagement in the platform via the number of instruments completed. Data quality was evaluated via standard attention checks. In Sample 5, we used the number of completed instruments as the main variable for comparing the social interaction component with the online-only experience.

The think-aloud interviews and focus group meetings (samples 1, 3, 4) were recorded and transcribed. We used thematic analysis with a flexible coding framework (17).

### 2.5 Ethical considerations

The project was approved by the Research Ethics Committee of the Institute (CAAE: 60974422.9.3001.0087). All participants electronically signed an Informed Consent Form, and the qualitative interviews were recorded with the participants' consent.

### 2.6 Data availability statement (DAS)

The datasets generated during the current study are available in the OSF repository (https://osf.io/yh7ae/).

## 3 Results

### 3.1 Engagement data considering all samples

**Figure 1:**
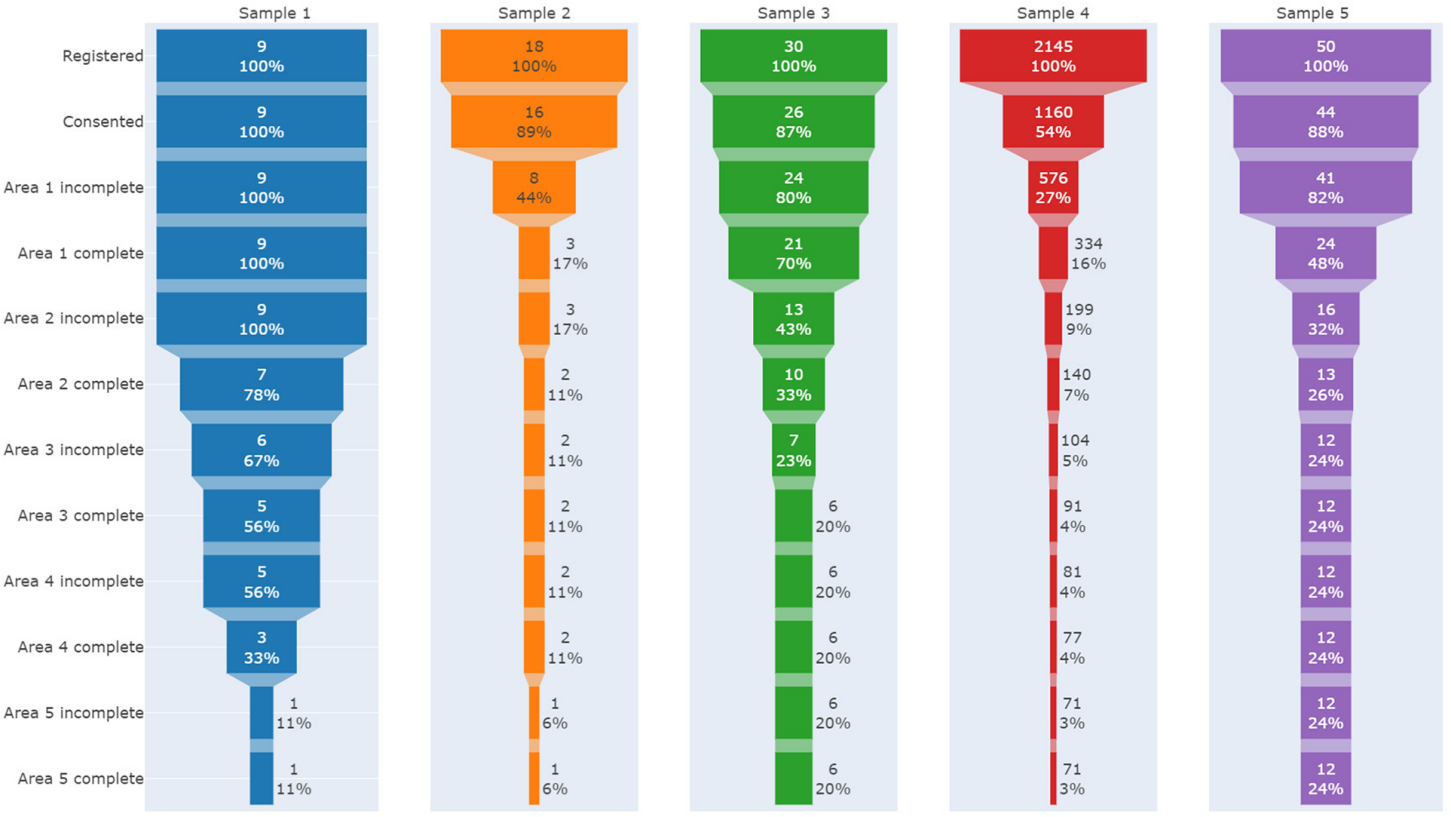
Funnel plots indicating participants drop out during the stages of the journey in each sample.

We observed substantial dropout rates across all samples during the usage of the journey, as evidenced by the low completion rates illustrated in Figure 1. This trend persisted even in samples where participants had increased interaction with the research team. Notably, data from Sample 4, which involved a public online launch of the platform, revealed a significant bottleneck at the early stages, specifically after signing the consent form and following the initial segment of the journey. This pattern suggests critical engagement challenges at the outset of the user experience, indicating the need for targeted interventions to improve initial user engagement and retention.

### 3.2 Online satisfaction survey

**Figure 2:**
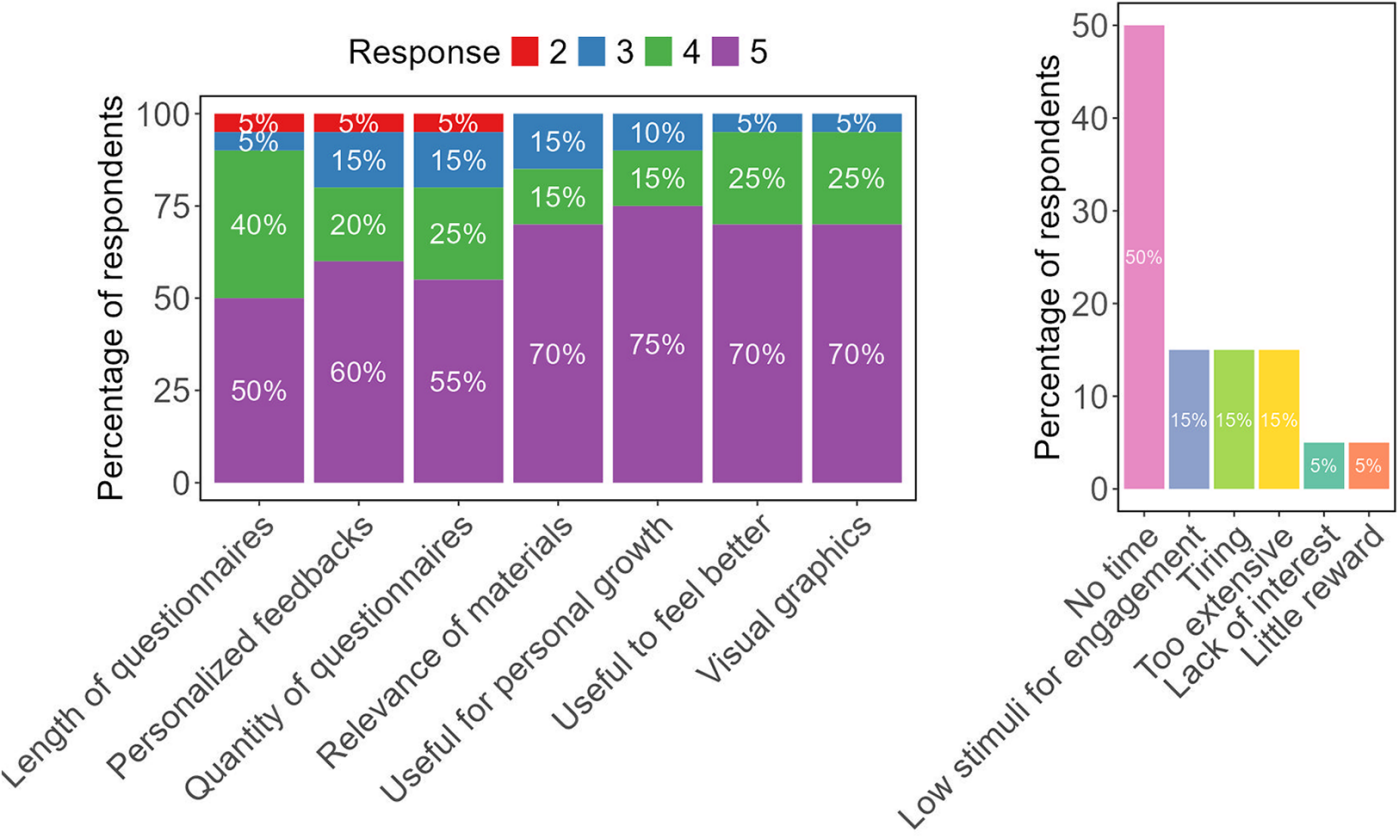
Satisfaction and adherence barriers questionnaires: **(A)** satisfaction with the journey was assessed on a 5-point Likert scale (1 = most negative evaluation; 5 = most positive evaluation) with responses from 20 participants across samples 1–3. Stacked bars represent the percentage of users selecting each score; **(B)** percentage of respondents indicating each adherence barrier as identified in the questionnaire.

The overall satisfaction in samples 1–3 was high (Figure 2A), with the mean across all questions being 4.5 (SD 0.76) on a 5-point Likert scale. The lowest mean was for the size of questionnaires, indicating somewhat lower satisfaction. The main disengagement factor was a lack of time, reported by 50% of respondents (Figure 2B). Further factors included the online journey being tiring, too extensive, and the lack of reminders (15% of respondents).

### 3.3 Online survey on facilitators and barriers to adherence

In this survey, we received responses from 27 participants. The satisfaction ratings were generally positive, with all mean scores exceeding the midpoint. Once again, the lowest satisfaction rating pertained to the number of instruments included (see Supplementary Figure S3).

**Figure 3:**
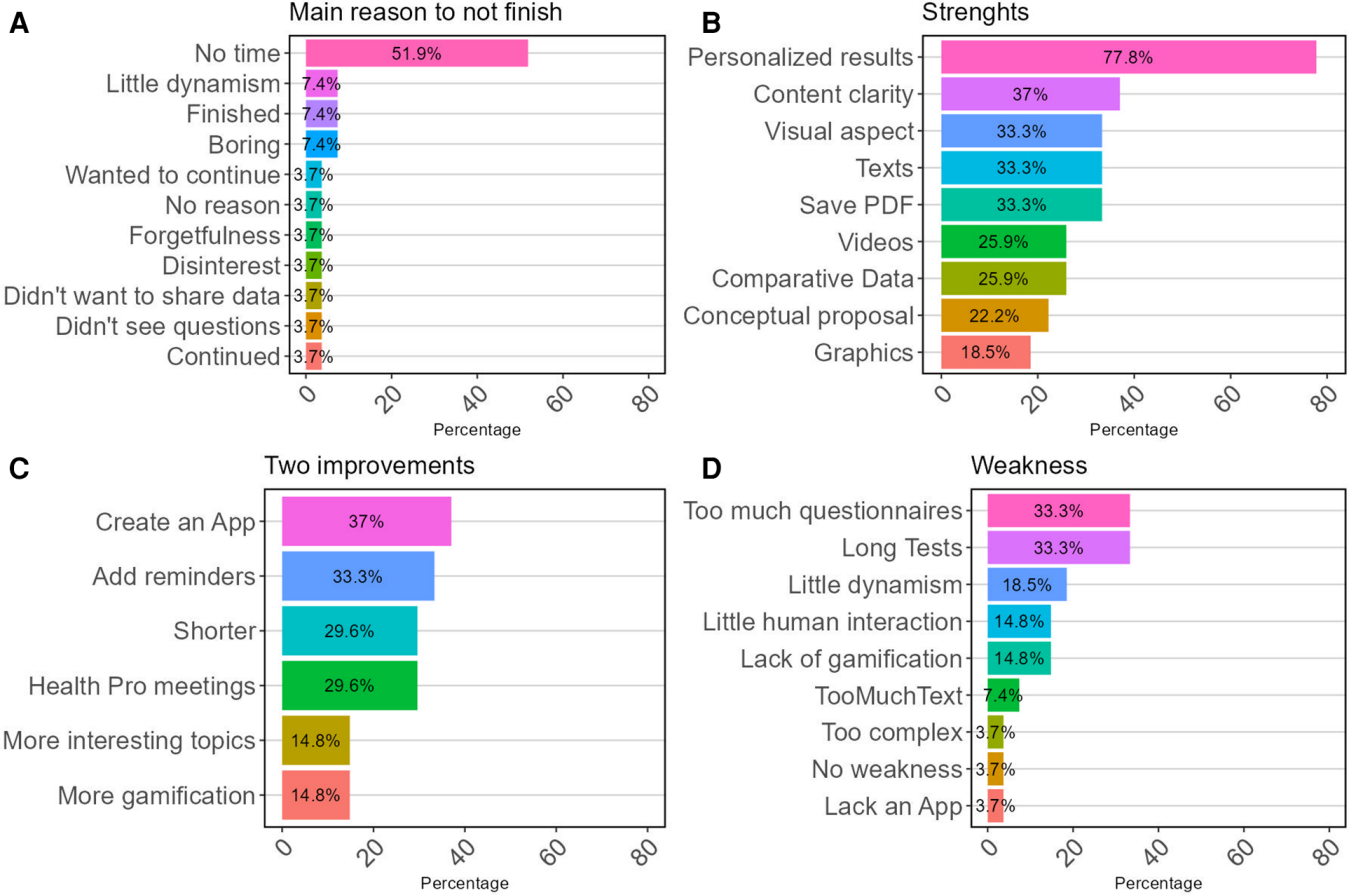
Main categories of the online survey on facilitators and barriers to adherence to the journey, when responding on **(A)** main reasons for non-completion; **(B)** strengths of the platform; **(C)** two suggestions for improvements; and **(D)** main weakness.

The predominant reason cited for non-completion of the platform's journey, as indicated in Figure 3A, was a lack of time. Despite this challenge, the platform's primary strength was identified as the provision of personalized results for each questionnaire. In terms of enhancements, participants predominantly recommended the development of a mobile application for the solution, the implementation of periodic reminders, a reduction in the overall length of the journey, and the inclusion of meetings with healthcare professionals. The principal criticisms from users focused on the excessive number and extensive length of the instruments required to be completed.

### 3.4 Focus group and think-aloud interviews

In the focus group, all participants completed at least one area of the journey (see Figure 1). Analyzing the reports of adherence difficulties from the focus group and think-aloud interviews, we identified five main themes of disengagement. Some reasons for disengagement were associated with incompatible time, lengthy questionnaires, long journey, lack of dynamism, and the necessity of a specific audience. The themes and exemplary responses are shown in Supplementary Figure S4.

Participants expressed significant overall satisfaction and a deep personal connection with the personalized feedback, as revealed in the excerpts from the focus group. For example, a 60-year-old female mentioned: “It's me entirely in all these here”, and a 19-year-old female noted: “I really identified with it. I liked the graphic, the way he scores, and the justifications that appear”.

### 3.5 Usage patterns in the fully self-guided mode

In the fully self-guided mode (sample 4), most users (88.9%) accessed the solution only on a single day and in a single session. We had a significant drop-out of participants (46.3%) between the registration on the platform and the consent form, with only 3.3% finishing all activities (see Figure 1).

### 3.6 Exploratory results on engagement: online experience vs. hybrid (online-in-person)

Participants in the hybrid group, which combined online and in-person elements, completed a higher number of instruments (mean: 7.68, SD: 7.08) compared to their counterparts (mean: 2.68, SD: 4.47) in the online-only group: *t* (40.533) = 2.98, *p* = .005, Δ_Glass_ = 0.71. Additionally, more individuals from the hybrid group completed all activities (40% of the 25 registered), compared to 8% in the online-only format (see Supplementary Figure S5).

## 4 Discussion

Our study revealed high overall satisfaction with the educational self-awareness journey, paralleled by noteworthy areas for enhancement. In line with similar research (8), we predominantly observed a one-time, concentrated use of the platform. This had a significant impact on the platform's usage plans. Initially, we envisioned it as a completely self-guided intervention for internet users, with clinical trials planned to verify its effectiveness in managing potential mental health conditions, such as anxiety. However, due to engagement difficulties and usage patterns concentrated on a single day, we concluded that the platform is more suitable as a mental health awareness mechanism or as a support tool for hybrid interventions led by health professionals. A considerable barrier in the digital-only format was the electronic consent form, which notably decreased access rates. It is important to highlight that in Brazil, the consent form needs to be a standardized document due to specific regulations for scientific studies involving sensitive data.

As shown in Figure 1, there is a clear disparity in engagement results from the initial phases of the journey. Consent rates vary significantly among different samples, with Samples 1–3, and 5 achieving consent rates above 87%, while Sample 4 (completely online) only achieved 54%. Both Sample 2 (that received a minimal in-person encouragement to register) and Sample 4 experienced rapid disengagement, with only 16% or 17% of participants completing the first stage out of five total steps. Sample 1, which had the most substantial in-person stimulus, demonstrated the highest initial adherence, with 78% of participants progressing to the halfway point of the intervention (Area 2 complete, which includes eight out of sixteen questionnaires). However, engagement declined in the later stages of the journey after face-to-face interaction ceased.

When evaluating the completeness of all stages of the journey, better and comparable results emerge, with approximately one in every four or five participants completing the entire process in Samples 5 and 3, respectively. It is noteworthy that in Sample 5, participants were invited in person, and a certificate of complementary hours possible to be used in the university curriculum was offered. Furthermore, half of Sample 5 received an extra personal meeting after two weeks. In Sample 3, there was a professional bound between researchers and participants, and three email reminders (push notifications) were sent to encourage engagement, which seem to have had a positive influence in adherence.

These insights can be useful for determining the appropriate timing for face-to-face contact in digital interventions in future studies. This approach may help achieve different objectives, such as enhancing initial adherence or improving completion rates of the proposed intervention. Based on our experience, an initial face-to-face meeting, followed by another personal meeting after a few weeks aiming at closing the intervention, seems to yield the best balance between the in-person effort required, engagement achievement, and intervention completion.

A recurring theme affecting participants’ engagement with the platform was the limited time availability. This aligns with suggestions that shorter activities might foster greater participant involvement (18). While visual feedback was provided, future versions may benefit from more image-centric and less text-heavy content to appeal to users (19). Aligned with previous research (12), the personalized results were one of the main strengths of the journey. This systematic review (12) also indicated four factors that positively impact adherence in digital interventions: personalization (or tailoring content to individual needs), push notifications, user-friendliness, and personal support complementary to the digital intervention. In terms of enhancements, participants predominantly recommended the development of a mobile application for the solution.

Participants also highlighted a lack of dynamism and gamification elements that induced disengagement. Improvement in these aspects is a salient strategy for enhancing retention in digital mental health solutions (20, 21). Persuasive features, such as self-monitoring, reminders, dialogue support, and social support, can also enhance the effectiveness of digital mental health interventions by encouraging users to engage in their chosen activities and promoting adherence (22). Therefore, the Persuasive System Design (PSD) framework (22) is highly recommended for similar internet-based interventions. In response to frequent suggestions, we plan to incorporate automated reminders in the next iteration of the platform, and we are exploring ways to integrate direct contact with healthcare professionals.

The exploratory findings of the study comparing online-only and hybrid interaction with the platform suggest an increased engagement in the hybrid model that included in-person meetings, supporting recent recommendations for hybrid approaches to boost user adherence and retention (23, 24). However, it is important to exercise caution when interpreting these results. Since this was not a structured experiment, the findings are preliminary and exploratory in nature. A formal, controlled study is necessary to ascertain the impact of hybrid mode more definitively vs. online-only modalities regarding users' engagement and completion rates in digital mental health interventions. It is also crucial to acknowledge the limitation of participant self-selection in their respective groups.

Despite the feasibility of digital mental health in LMICs, integrating DMHIs into healthcare professionals' routines remains disappointingly low (25). Their involvement could make new technologies more relevant, improving userś engagement outcomes. We advocate for further research to determine the most effective ways to deliver hybrid care models (7, 23). As presented by other authors (7), analyzing users' characteristics may be a valuable area for future studies to determine the most effective formats to offer. Clinical trials evaluating the impact of different in-person and online stimuli on adherence to digital interventions can also help identify the best hybrid and remote arrangements for future interventions. Exploring the impact of minimal in-person stimuli on adherence to DMHIs remains a research frontier that could be addressed by other studies. Our findings show potential in delivering personalized mental health information yet highlight enduring challenges in a solely digital environment.

## Data Availability

The original contributions presented in the study are included in the article/Supplementary Materials, further inquiries can be directed to the corresponding author.
