# Supplementary material for: Engagement challenges in digital mental health programs: hybrid approaches and user retention of an online self-knowledge journey in Brazil
Source: Front Digit Health. 2024 Sep 25;6:1383999. doi: 10.3389/fdgth.2024.1383999 (PMC11461457; doi:10.3389/fdgth.2024.1383999)
Supplement: Supplementary file 1 [file Image1.pdf]

Thanks for your answers! **This test measured some personality traits**. Science has discovered five dimensions or traits of human personality that can be used to describe our behaviour. These **five dimensions** can manifest themselves in different ways, being stronger or weaker in each person. Considering all dimensions, a unique combination is formed that is our personality.

To better understand the 5 dimensions of your personality, check out your complete profile now.

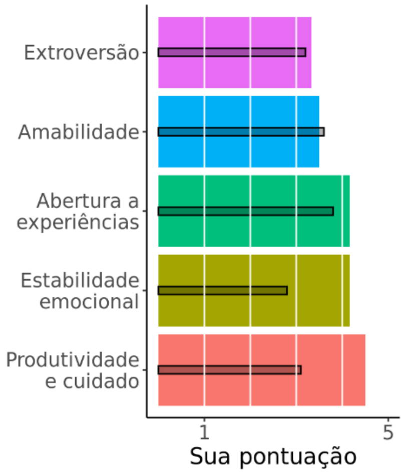

Coloured bars indicate your score. Black bars indicate the population mean.
Now, see below a personalized description of each dimension:

Extroversão (Extroversion)

This characteristic concerns interpersonal relationships and where you get your energy from, or how you seek to engage with the outside world. Your score was higher than most people. According to this score, it may be that you really enjoy social contexts, talking to others and always seek social contact. People with this score like to engage with the outside world and are action oriented. In other words, they are people who accept the group's ideas, like to talk, and interact and have a lot of energy.

Amabilidade (Agreeableness)

This trait talks about the tendency to be sociable and warm, seek harmony in relationships and put the interests of others before one's own interests or not. Your score is below the population average. Its results are more frequent in people who are a little more critical, who speak directly what they think, or who are perhaps a little more reserved. Sometimes being skeptical about the intentions of others can make you a little suspicious and thus generate a preference to act in your own interests to make sure you don't end up getting hurt.

(…)

Your strongest trait is:
"Produtividade e cuidado"

Your second strongest trait is:
"Abertura a experiências"

And now?

It is important to remember that there are no good or bad scores. Each score brings with it characteristics that may be more positive in one context and less in others (...)

Our personality traits, despite being relatively stable for most of our adult lives, **undergo changes and are sensitive to our efforts.**

So, what did you think of your profile? Did you think it describes you well? Are you happy with your personality traits or do you want to work on changing some parts?

Keep going on the journey to get to know yourself more and more.
