# Supplementary material for: Engagement challenges in digital mental health programs: hybrid approaches and user retention of an online self-knowledge journey in Brazil
Source: Front Digit Health. 2024 Sep 25;6:1383999. doi: 10.3389/fdgth.2024.1383999 (PMC11461457; doi:10.3389/fdgth.2024.1383999)
Supplement: Supplementary file 4 [file Image4.pdf]

Supplementary Material

Engagement challenges in digital mental health programs: hybrid approaches and user retention of an online self-knowledge journey in Brazil

Felipe Azevedo Moretti[1*†], Tiago Soares Bortolini[2†], Larissa Marques Hartle[2], Ronald Fischer[1]

*** Correspondence:** Felipe Azevedo Moretti: felipe.moretti@idor.org

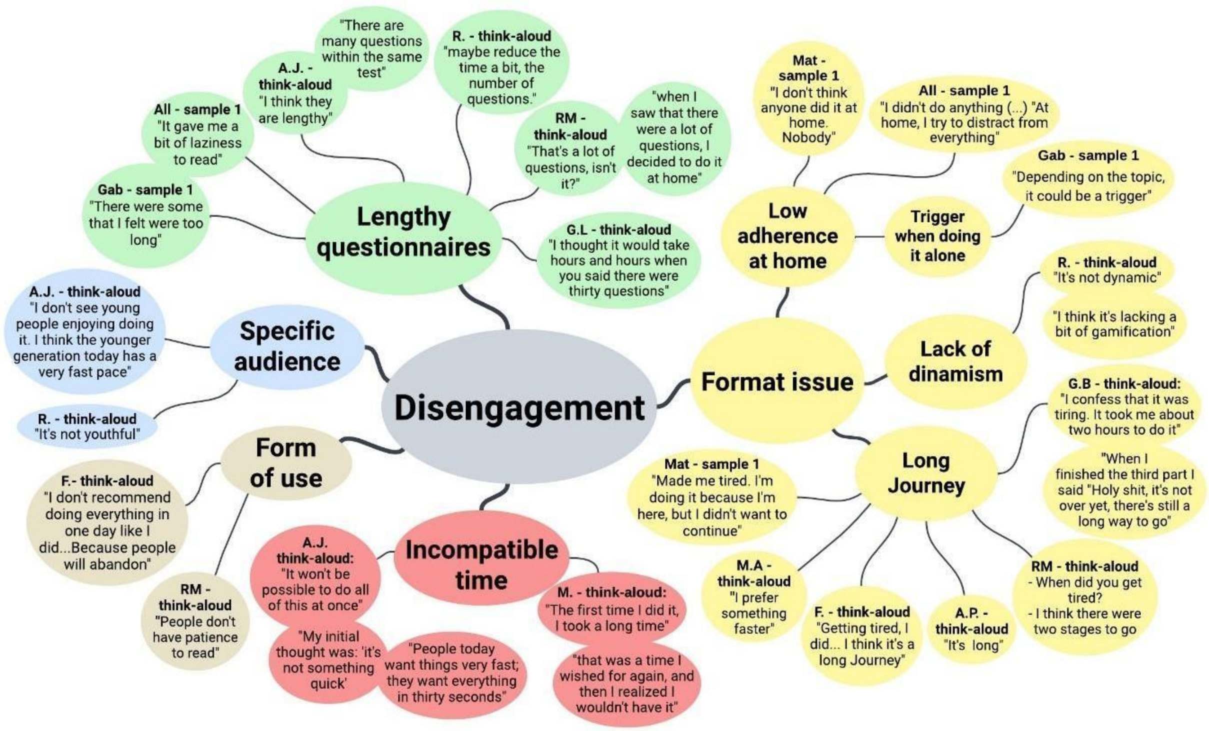

Supplementary Figure 4. Mind map of disengagement factors associated with the self-knowledge journey. The sample's socio-demographic information is as follows: F. (46 years old, female, human resources analyst); A.J. (44 years old, female, content producer); M. (41, female, researcher); A.P. (38, female, health and safety engineer); R. (33, male, engineer); M.A (33, female, financial analyst); RM (29, female, institutional agent); G.L. (21, saleswoman); GB (18, female, student); Gab (18, male, IT trainee); Mat (27, male, IT instructor); All (20, male, student)
