# Supplementary material for: Engagement challenges in digital mental health programs: hybrid approaches and user retention of an online self-knowledge journey in Brazil
Source: Front Digit Health. 2024 Sep 25;6:1383999. doi: 10.3389/fdgth.2024.1383999 (PMC11461457; doi:10.3389/fdgth.2024.1383999)
Supplement: Supplementary file 5 [file Image5.pdf]

Supplementary Material

Engagement challenges in digital mental health programs: hybrid approaches and user retention of an online self-knowledge journey in Brazil

Felipe Azevedo Moretti[1*†], Tiago Soares Bortolini[2†], Larissa Marques Hartle[2], Ronald Fischer[1]

*** Correspondence:** Felipe Azevedo Moretti: felipe.moretti@idor.org

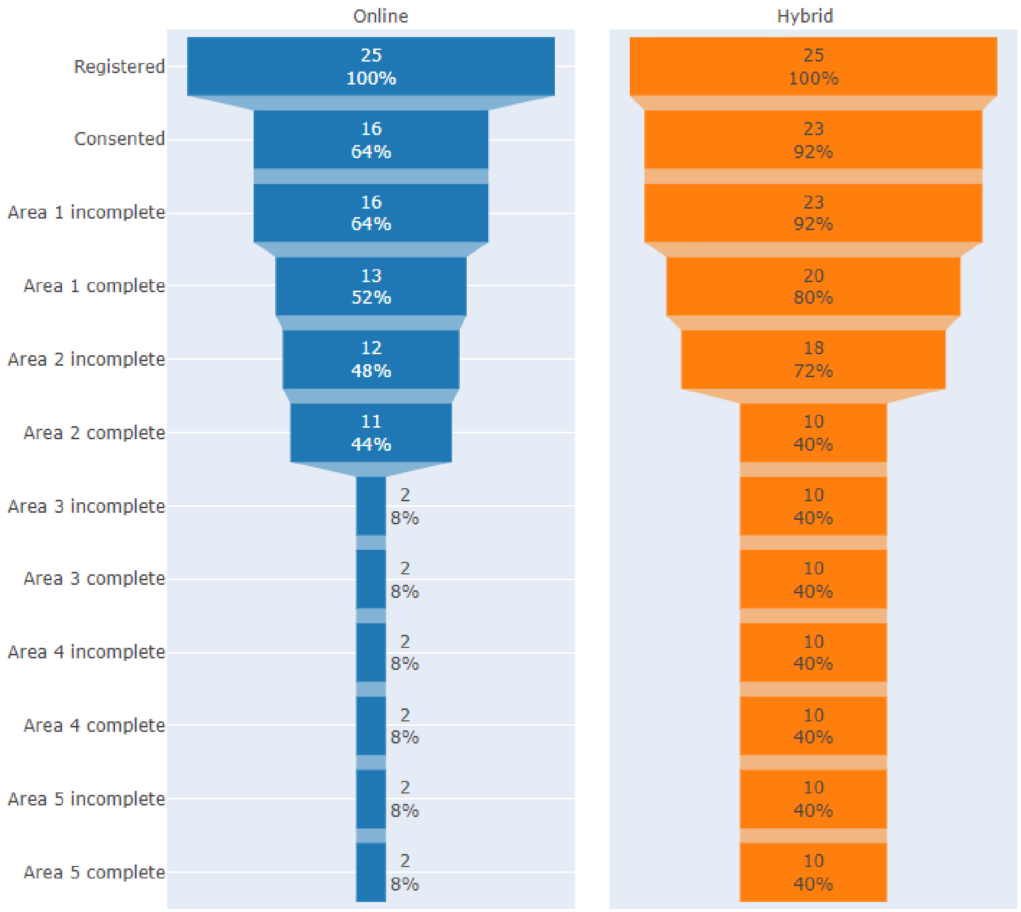

Supplementary Figure 5. Progress in the journey of sample 5, comparing people who did only online (n=25) vs. hybrid mode (n=25).
